# The Relationship of Perforated Appendicitis with Total and Direct Bilirubin

**DOI:** 10.7759/cureus.6326

**Published:** 2019-12-08

**Authors:** Murat Kanlioz, Turgay Karatas

**Affiliations:** 1 General Surgery, Beylikdüzü Kolan Hospital, Istanbul, TUR; 2 Anatomy, Inönü University, Malatya, TUR

**Keywords:** perforated appendicitis, total bilirubin, direct bilirubin, acute appendicitis, acute appendicitis

## Abstract

Introduction

Very different results have been reported regarding the relationship between bilirubin and perforated appendicitis. We observed this relationship with our own studies.

Methods

The patients, who underwent appendectomy, were retrospectively categorized as perforated and non-perforated based on their files. Those with a total bilirubin (TB) 1.20 mg/dL or less were considered normal whereas those with a 1.21 mg/dL or higher were considered having a high. Those with a direct bilirubin (DB) 0.50 mg/dL or less were considered normal whereas those with a 0.51 mg/dL or higher were considered having a high. The patients were assessed under two groups. Perforated appendicitis (PA) and non-perforated appendicitis (NPA) were analyzed according to the TB in Group 1 and the DB in Group 2.

Results

Group 1 included 269 patients whose TB were measured. Of those, 218 had NPA and 51 had PA. The rate of patients with high TB among the patients with PA was 1.37 times higher than those with NPA (p ˂ 0.01). Group 2 included 258 patients whose DB values were measured. Of those, 208 had NPA and 50 had PA. The rate of patients with high TB among the patients with PA was 1.71 times higher than those with NPA (p ˂ 0.001).

Conclusion

In the diagnosis of PA, both TB and DB show low diagnostic values. In the diagnosis, they can only be considered as a supportive factor to other parameters. However, in the case of a differential diagnosis, we recommend using DB since it has a higher sensitivity and specificity.

## Introduction

Acute appendicitis (AP) is the most common non-traumatic surgery emergency [1]. It is important to determine whether the AP is perforated or not at the stage of diagnosis to minimize complications [2]. The process in AP begins with the obstruction of the appendix lumen. Lymphoid activity is important in lumen blockage. AP is most common in ages 20-30, and it is known that lymphoid activity is high in this period [3]. Other factors in the obstruction of the appendix lumen include fecaloma, parasitosis, foreign bodies and rarely tumoral formations. As the drainage of the mucus produced from the mucosa cannot occur due to the blockage of the lumen, the pressure inside the lumen will increase. As the pressure increases, venous and arterial blood flow is disrupted, microbial colonization increases and pain is added to the table, respectively [4]. Perforation may be seen in the progressive process. This is attributed to the increase of Kupffer cell function and hepatocyte injury as a result of excessive bacterial and toxin load from the liver via portal venous system, as well as increased serum bilirubin and suppression of hepatic bilirubin destruction functions as a result of increased acute phase reactants such as IL-6 and TNF [5]. Anamnesis is important in the diagnosis of AP. The patient applies with anorexia, nausea, abdominal pain and fever [3]. In the examination, defense and rebound tenderness are typical in the lower right quadrant of the abdomen [4]. Some maneuver and findings are helpful in the diagnosis (Rovsing's sign, etc.). Leukocytosis, increased neutrophil rate, increased C-reactive protein (CRP), increased bilirubin, procalcitonin and IL-6 are helpful in diagnosis [1,6-8]. Ultrasonography, computed tomography and magnetic resonance imaging are valuable in diagnosis [2,9]. It is important to observe a curve of the bowel with edema in the right lower quadrant in X-ray. Some scoring tests (Alvarado, Heidelberg) and laparoscopy are used in the diagnosis [10-12]. Primary treatment in AP is surgery. However, there are also studies evaluating medical treatment as an option [13]. In the surgical treatment, laparoscopic and open surgery are performed to investigate the importance of total bilirubin (TB) and direct bilirubin (DB) levels in the diagnosis of perforated appendicitis (PA) [10].

## Materials and methods

The files of the 269 patients, who underwent appendectomy, were examined retrospectively. Patients who were reported as acute appendicitis as a result of post-operative pathology were included in the study. The patients with complete results of pre-operative serum bilirubin and post-operative pathology were included in the study. The pre-operative serum TB and DB levels of the patients were recorded. Those with a TB 1.20 mg/dL or less were considered normal whereas those with a 1.21 mg/dL or higher were considered having a high. Those with a DB 0.50 mg/dL or less were considered normal whereas those with a 0.51 mg/dL or higher were considered having a high. The patients were categorized as “perforated appendicitis (PA)” and “non-perforated appendicitis (NPA)” based on their post-operative pathologic results. The patients were assessed under two groups. It was analyzed whether there is a difference between the PA and NPA according to the TB in Group 1 and DB in Group 2. SPSS statistical software was used. The between-groups distribution was evaluated using Kolmogorov-Smirnov test. The results were analyzed using “Pearson Chi-Square Test”. p ˂ 0.05 was considered significant.

## Results

In Group 1, the pre-operative TB in 269 patients were measured. The patients, of whom 148 (55%) were male and 121 (45%) were female, had a mean age of 28 years. Of those patients, 218 (81%) had NPA and 51 (19%) had PA. TB was high in 31 (14%) of 218 patients with NPA, and in 10 (20%) of 51 patients with PA (Table 1). TB was 1.37 times higher in the patients with PA than those with NPA (p ˂ 0.01). In patients with PA, the TB showed a sensitivity of 20%, a specificity of 14%, a positive predictive value of 24%, and a negative predictive value of 18%.

**Table 1:** Total bilirubin levels in appendectomies

	Total Bilirubin			Total
	Normal (n)		High (n)	
Non-perforated	187 (86%)	31 (14%)		218
Perforated	41 (80%)	10 (20%)		51
TOTAL	228 (85%)	41 (15%)		269

In Group 2, DB in 258 patients were measured in the pre-operative period. The mean age of the patients, of whom 144 (56%) were male and 114 (44%) were female, was 28.7 years. Of those patients, 208 (81%) had NPA and 50 (19%) had PA. DB was high in 17 (8.17%) of 208 patients with NPA, and in seven (14%) of 50 patients with PA (Table 2). DB was 1.71 times higher in the patients with PA than those with NPA (p ˂ 0.001). DB showed a sensitivity of 14%, a specificity of 8%, a positive predictive value of 29%, and a negative predictive value of 18%.

**Table 2:** Direct bilirubin levels in appendectomies

	Direct Bilirubin			Total
	Normal (n)		High (n)	
Non-perforated	191 (92%)	17 (8%)		208
Perforated	43 (86%)	7 (14%)		50
TOTAL	234 (85%)	24 (15%)		258

In the analysis performed using Kolmogorov-Smirnov test, between-groups distribution was considered normal.

## Discussion

There are many methods used in the diagnosis of acute appendicitis and while determining whether it is perforated or not following the diagnosis. One of those methods includes measuring the serum bilirubin level. This study aims to reveal the correlation between the bilirubin level and the appendicitis. The methods used during the diagnoses are aimed at ensuring an early diagnosis and recognizing the perforation, if any. Because, a perforation in appendicitis leads to a significant increase in morbidity and mortality. The aim is to perform the surgery before the appendicitis gets perforated. Besides, if diagnosed with PA, the patient should be given priority in the treatment and the processes should be accelerated. In both study groups, the perforation rate was 19%. The perforation rates in appendectomies performed were reported to be 20.2% in the study carried out by Barreto et al., 15.7% in the study of Siddique et al., and 24% in the study of Adams and Jaunoo [7,8,14]. In our study, there was 15% hyperbilirubinemia in Group 1. In the study of Estrada et al., 59 (38%) of the 157 patients who underwent appendectomy had hyperbilirubinemia [15]. Bonadio et al. reported that TB was high in 22% of the NPA [16]. In our study, this rate was 14.22% in patients with NPA in Group 1. The rates of high TB in PA were reported to be 36% by Bonadio et al. and 48% by Müller et al., whereas Saxena et al. reported that this was 100% in their study [16-18]. However, we found in our study that the total bilirubin level was high in 20% of PA in Group 1. We believe that especially the rate reported by Saxena et al. is highly disputable. Alanis-Rivera et al. reported in their study that the rate of TB in patients with PA was 17 times higher than in those with NPA [19]. This rate was reported to be five times higher by Eren et al. [20]. In the 557-patient series of Adams and Jaunoo, no significant difference was found between the perforated and non-perforated group in terms of bilirubin values [14]. In Group 1, we found that the TB in patients with PA was 1.37 times higher than those with NPA. The fact that such differing results have been obtained so far requires undertaking more detailed studies.

Eren et al. reported that the DB was 36 times higher in patients with PA than in those with NPA [20]. In another study, however, Nevler et al. set forth that they found a significant correlation between PA and the DB (p ˂ 0.001) [21]. In Group 2, we found that DB in patients with PA was 1.71 times higher than in those with NPA.

Among the appendectomies, our results in terms of PA are at an average level according to the literature, however it is clear that these rates need to be further reduced. In our age, the fact that one out of every five cases is perforated and that the perforation leads to severe mortality and morbidity rates are unacceptable. Many studies have so far reported that there is a correlation between PA and bilirubin, however there are serious differences between the results obtained. Whilst some studies show a strong correlation in between, our studies do not support such strong correlation. When we examine the level of bilirubin in appendicitis in terms of sensitivity, specificity, and predictivity, we see, on the basis of our findings, that these constitute a supportive factor for the diagnosis together with several parameters.

## Conclusions

In PA, we can use the bilirubin levels for the purpose of differential diagnosis. However, we should note at this point that, if the bilirubin level is high, this is of significance in terms of diagnosis, however a non-high bilirubin level should not mislead us that there is no perforation. Given its sensitivity and specificity, we can state that DB has a higher diagnostic value than TB.
